# The Effect of Race, Sex, and Insurance Status on Time-to-Listing Decisions for Liver Transplantation

**DOI:** 10.1155/2010/467976

**Published:** 2010-12-23

**Authors:** Cindy L. Bryce, Chung-Chou Ho Chang, Derek C. Angus, Robert M. Arnold, Maxwell Farrell, Mark S. Roberts

**Affiliations:** ^1^Division of General Internal Medicine, Department of Medicine, School of Medicine, University of Pittsburgh, Pittsburgh, PA 15213, USA; ^2^Section of Decision Sciences and Clinical Systems Modeling, University of Pittsburgh, Pittsburgh, PA 15213, USA; ^3^Department of Health Policy and Management, Graduate School of Public Health, University of Pittsburgh, Pittsburgh, PA 15213, USA; ^4^Department of Biostatistics, Graduate School of Public Health, University of Pittsburgh, Pittsburgh, PA 15213, USA; ^5^The Clinical Research Investigation and Systems Modeling of Acute Illness (CRISMA) Laboratory, Department of Critical Care Medicine, School of Medicine, University of Pittsburgh, Pittsburgh, PA 15213, USA

## Abstract

Fair allocation of organs to candidates listed for transplantation is fundamental to organ-donation policies. Processes leading to listing decisions are neither regulated nor understood. We explored whether patient characteristics affected timeliness of listing using population-based data on 144,507 adults hospitalized with liver-related disease in Pennsylvania. We linked hospitalizations to other secondary data and found 3,071 listed for transplants, 1,537 received transplants, and 57,020 died. Among candidates, 61% (*n* = 1,879) and 85.5% (*n* = 2,626) were listed within 1 and 3 years of diagnosis; 26.7% (*n* = 1,130) and 95% (*n* = 1,468) of recipients were transplanted within 1 and 3 years of listing. Using competing-risks models, we found few overall differences by sex, but both black patients and those insured by Medicare and Medicaid (combined) waited longer before being listed. Patients with combined Medicare and Medicaid insurance, as well as those with Medicaid alone, were also more likely to die without ever being listed. Once listed, the time to transplant was slightly longer for women, but it did not differ by race/ethnicity or insurance. The early time period from diagnosis to listing for liver transplantation reveals unwanted variation related to demographics that jeopardizes overall fairness of organ allocation and needs to be further explored.

## 1. Introduction 

Because the demand for transplant services has always exceeded the supply of donor organs, the transplant community as well as policymakers have long recognized the need to ensure that the organ allocation system is efficient and equitable [1–6]. The United Network for Organ Sharing (UNOS), which oversees waitlisting and allocation guidelines in the United States, indicates that access to organs will not be based on “political influence, race, gender, religion, or financial or social status” [7]. Yet, as noted by the Institute of Medicine, the transplant process involves numerous steps and inequities can take place anywhere along the way [8].

Historically, in cases where empirical data have shown systematic differences in waiting times or in the chances of receiving a transplant, UNOS has changed its policies to improve organ allocation procedures. Examples of changes include the institution of less stringent HLA-matching requirements for renal transplantation [9], the adoption of the final rule [6], and the use of the model of end-stage liver disease (MELD) scoring system for liver transplantation [10, 11]. For renal transplantation, researchers have access to population-based data about the early steps of the transplant process from the US Renal Data System [12–14]. But for other types of solid organ transplantation, including liver transplantation, information about the early steps is generally unavailable, so oversight is restricted to steps after listing. 

We linked several secondary data sources to identify a statewide, population-based cohort of patients with liver-related conditions and followed the cohort through the following stages of the transplant process: disease occurrence (incidence), disease progression (natural history), disease diagnosis, referral, and evaluation by a transplant center, placement on the transplant waiting list (listing), and receipt of an organ (transplantation). We previously reported that demographics were important in determining the likelihood that patients with liver disease would be able to access the transplantation process for evaluation and listing, but not in affecting the likelihood that they would undergo transplantation once they were listed [15]. This initial analysis evaluated only whether or not patients progressed to specific stages of the transplantation process; because of missing data, it did not address matters related to timing and timeliness.

In the current paper, we estimated the relationship between sociodemographics and the time required for patients to reach specific stages of the process. Specifically, we examined waiting times experienced by subsets of patients during 2 time periods. The subsets were based on gender, race/ethnicity, and insurance status. One period was the time between a patient's diagnosis of liver disease and his or her placement on the UNOS waiting list (an interval in which there is no formal oversight or centralized data collection effort), and the other period was the time between a patient's placement on the UNOS waiting list and his or her receipt of a transplant (an interval in which there is oversight). Our main hypothesis was that gender, race/ethnicity, and insurance status would be associated with variation in waiting times before, but not after, placement on the transplant waiting list.

## 2. Methods

### 2.1. Data Sources and Data Management

Our conceptual framework, data sources, and patient cohort have been described previously [15]. Briefly, we considered the stages in which a patient developed liver disease, was diagnosed, was referred to a transplant center and evaluated, was listed, and received a transplant. We assumed that most individuals who become sufficiently ill to be considered for a transplant were hospitalized at some point in their illness and, therefore, used hospital discharge data from the Pennsylvania Health Care Cost Containment Council (PHC4) to identify patients who had “liver-transplant potential.” Every nongovernment hospital in Pennsylvania is required by state law to submit clinically abstracted data to the PHC4 for all hospital discharges, and the accuracy of the data has been validated against chart reviews [16].

The PHC4 listed 310 participating hospitals statewide during the study period. It provided us with data for all patients with liver-related stays between 1994 and 2001. These data included the patients' age, gender, race/ethnicity, and county and ZIP code of residence, type of admission, admission and discharge diagnoses, procedures, and diagnosis-related group (DRG) codes, discharge destination, and Medical Illness Severity Grouping System (MEDISGRPS) disease category (mortality risk) and severity score. To help identify and classify patients with liver disease, we developed a detailed list of diagnostic and procedural codes related to liver problems. Based on our previous work [17], we classified patients in terms of 9 major categories of disease: viral hepatitis, alcoholic liver disease, autoimmune disorder, metabolic disease, primary sclerosing cholangitis, cancer, primary biliary cirrhosis, other chronic liver disease, and acute liver failure. 

We excluded patients who were discharged in 1994 as well as those who had previously received a liver-transplant or been listed for one. This left us with a cohort of 144,507 patients with liver disease from 272 hospitals that had been newly diagnosed between 1995 and 2001. We linked the index hospitalization records of these patients to the following: the liver referral and evaluation data from the 5 predominant liver-transplant centers in Pennsylvania (Albert Einstein Medical Center, Hospital University of Pennsylvania, Thomas Jefferson University Hospital, University of Pittsburgh Medical Center, and the VA Pittsburgh Healthcare System), the listing, allocation, and transplant data from UNOS, and the mortality data from the Bureau of Health Statistics and Research of the Pennsylvania Department of Health. 

For many patients in these data sets, the specific time at which some stages were reached was missing. However, the data were complete for the time of disease diagnosis, waitlisting, and transplantation. This allowed us to determine whether patients progressed to a subsequent stage and to measure the time intervals, in days, from diagnosis to listing (early waiting time) and from listing to receipt of a transplant (later waiting time). In each case, there were 3 possible outcomes: proceed to the subsequent stage of the process, remain at the current stage, or die.

The unit of analysis was the patient (not the registration). Although the data were deidentified by the honest broker, multiple listings could be identified and reconciled using a pseudoidentifier for patients. We combined the dates from all of the data sources to “timestamp” the patient's progression through the transplantation process, starting with index hospitalization (i.e., diagnosis) until either the earliest definitive outcome (i.e., transplant, death) or the end of the study period (i.e., still waiting).

In pooling the data sources, we applied 2 strategies for creating the longitudinal records for our patient cohort. First, we did not adjust the waiting times to account for periods when the patient was inactive (a special status category for patients on the waiting list), which increases the estimates of later waiting times from listing to transplantation. Second, for patients with multiple listings, we took the earliest listing date available. Both of these conventions minimize early waiting time from diagnosis until listing and maximize later waiting times from listing to transplant, serving to bias against our hypothesis that early waiting times vary with socioeconomic variables (but later waiting times do not).

Our study was funded by the National Institute of Diabetes and Digestive and Kidney Diseases and approved by the institutional review boards at the University of Pittsburgh and other participating transplant centers. We protected patient confidentiality by having the PHC4 serve as an honest broker to link records across data sources and provide our team with deidentified versions of the files.

### 2.2. Statistical Analyses

To characterize patients in terms of sociodemographic and clinical data, stage of the allocation process (diagnosis, listing, and receipt of a transplant), waiting times, and outcomes, we used descriptive statistics. 

To compare the characteristics of subsets of patients at each stage of the process, we used univariable and multivariable survival models that included the following covariates: age, gender, race/ethnicity (white, black, Hispanic/Asian/other, and unknown), insurance status (commercial only, Medicaid only, Medicare only, combined Medicare plus commercial, combined Medicare plus Medicaid, uninsured, and unknown) based on the index hospitalization, type of liver disease using the diagnostic categories listed above, and severity of illness at the time of diagnosis, based on the MediQual severity scale [18] and ranging from 0 to 4 (representing none, minimal, moderate, severe, or maximal) or coded as unknown. Given our focus on state-level data, there was no variation in terms of geographic region, but we did include location of transplant center (Pennsylvania versus non-Pennsylvania) as a covariate in the models to account for Pennsylvania residents who were listed and/or transplanted at other centers. We included year of index hospitalization and also tested for interaction variables (e.g., interaction between diagnosis and gender). 

In the unadjusted case, we looked at differences in early waiting times based on the proportion of *diagnosed* patients who were placed on the transplantation waiting list within a specified period (i.e., 1, 3, 5, and 8 years) using Kalbfleisch and Prentice's cumulative incidence technique [19]. Similarly, we looked at differences in later waiting times by examining the proportion of *listed* patients who received transplants at these same intervals. In both cases, we controlled for the competing risk of death.

In the adjusted models, we tested for differences in early waiting times by estimating the time to listing for the entire cohort, and we tested for differences in later waiting times by estimating time to transplant for patients on the waiting list. We used Fine and Gray's survival model that takes death and other competing risks into account [20]. We compared differences in the magnitude and significance of our primary covariates (gender, race/ethnicity, and insurance status) while adjusting for the other covariates.

We retained all covariates with *P* < .05, and we used SAS, version 9.2 (SAS Institute Inc., Cary, NC) and STATA, version 8 (Stata Corp., College Station, TX) for all analyses.

## 3. Results

Table 1 shows data for 3 groups—the full cohort, the subset of patients listed for transplantation, and the final subset of patients who received transplants—stratified by sociodemographic and clinical characteristics. In all 3 groups, the largest proportions of patients were male, were 40–64 years of age, had commercial health insurance only, had a diagnosis of viral hepatitis, alcoholic liver disease, or autoimmune disorder, and had a moderate or severe level of illness. Because of the large sample size, between-group differences were statistically significant for all covariates. In general, however, differences between the first and second groups (full cohort and listed patients) were larger than differences between the second and third groups (listed patients and transplant recipients).

Of the 144,507 adults in our final cohort of patients with liver-transplant potential, 3,071 (2.1%) were placed on the transplant waiting list. Of these 3,071 patients, 1,537 (50.0%) went on to receive a liver transplant by December 2003. Based on raw waiting times, 61% (*n* = 1,879) of all transplant candidates were placed on the waiting list within 1 year of diagnosis, and 85.5% (*n* = 2,626) were listed within 3 years of diagnosis. Among transplant recipients, 26.7% (*n* = 1,130) received transplants within 1 year of listing and 95% (*n* = 1,468) received transplants within 3 years of listing. A total of 57,020 patients (39.5%) died during the study period.

### 3.1. Univariable Analyses

Because waiting times are censored for patients who do not progress to the next stage of the process (e.g., diagnosed patients who are not listed, and listed patients who do not receive transplants), mean waiting times cannot be computed. Instead, Table 2 reports the cumulative percentage of patients who progressed to the next stage of the transplantation process within specific time intervals, first among diagnosed patients and then among listed patients, as well as the cumulative percentage of patients who died (the competing risk) during those same intervals.

Among the 144,507 diagnosed patients in the cohort, 1.3% were listed within 1 year of the index hospitalization and 2.1% were listed within the 8-year study period (Table 2, top panel). During the same period, 21.9% of the patients died within 1 year and 38.6% died within 8 years. In the stratified results, women diagnosed with liver disease were less likely than their male counterparts to be listed but were also less likely to die. At every interval, black patients had lower probabilities of being listed, and patients in the Hispanic/Asian/other category had lower probabilities of death than the overall cohort did. In terms of insurance status, patients with commercial insurance were more likely to be listed and less likely to die whereas patients with any form of Medicare insurance coverage exhibited the opposite pattern and were less likely to be listed and more likely to die than the overall cohort.

For later waiting times after listing (until transplantation) (Table 2, bottom panel), women continued to receive transplants at lower rates than men and had greater risks of dying on the waiting list. Black patients showed the same pattern, with fewer transplants and more deaths than white patients. Both the Medicare patients and the uninsured patients (including self-pay patients) were listed at higher-than-average rates, but the Medicare patients had higher-than-average risks of death while the uninsured patients had lower-than-average risks of death.

### 3.2. Multivariable Analyses

The adjusted competing risks models included the primary covariates (gender, race/ethnicity, and insurance status), all other covariates mentioned above, interaction terms for diagnosis and gender, and interaction terms for diagnosis and race/ethnicity for both the early waiting period and the later waiting period models. The main effect of insurance status was nonsignificant in the later period (after listing), so interaction terms between diagnosis and insurance were only included in the model to estimate early waiting times. Full regression results for the models are provided in Table 3.

**Figure 1:**
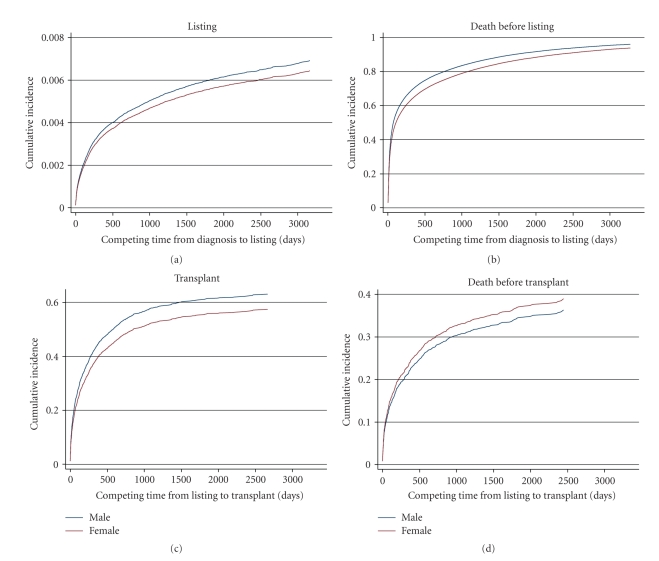
Gender-related results in the adjusted competing risks model.

**Figure 2:**
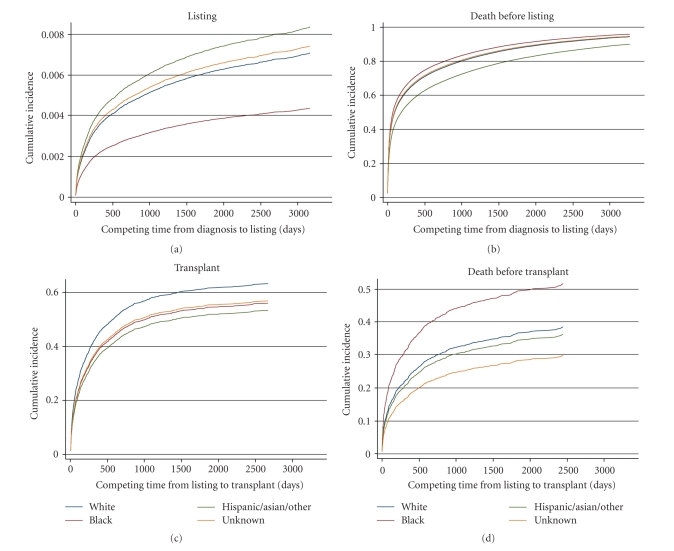
Race/ethnicity-based results in the adjusted competing risks model.

**Figure 3:**
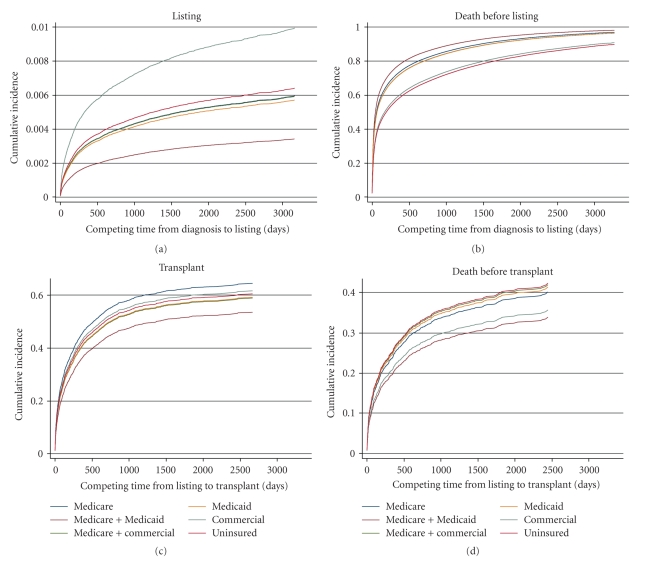
Insurance-based results in the adjusted competing risks model.

**Figure 4:**
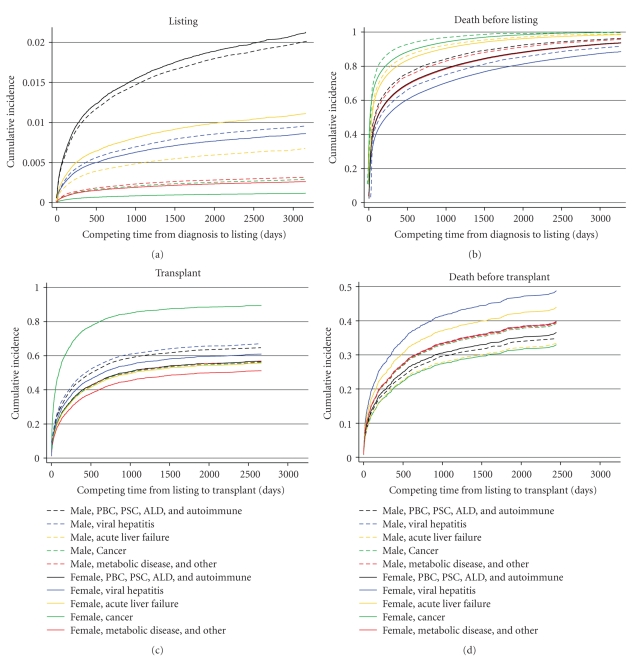
Gender-related results in the adjusted competing risks model. Abbreviations: PBC-primary biliary cirrhosis; PSC-primary sclerosing cholangitis; ALD-alcoholic liver disease.

 Graphs of the findings for gender, race/ethnicity, and insurance status are shown in Figures 1, 2, and 3, respectively. The information is analogous to that presented in Table 2 for the univariable analyses. Of note, in competing risk models, if the longest noncensored followup time in the observed data set coincides with a patient death, then the estimated cumulative incidence function for death (i.e., the competing risk) converges to 1.0 in the graph. This is the case for all of the competing risk graphs (right-hand side) in Figure 1 through 3; this artifact is similar to the way in which Kaplan-Meier graphs show no survivors at the end of the followup period. Diagnosis-specific interaction results for gender are provided in Figure 4.

#### 3.2.1. Gender

In terms of the main effect of gender, early waiting times (from diagnosis to listing) were similar for men and women (beta = 0.052, *P* = .31; Table 3), and men were more likely to die without ever being listed (beta = −0.2175, *P* < .0001). 

However, the overall effect of gender on early waiting time was slightly different when the interactions between gender and diagnosis and the distribution of men and women in the diagnosis categories were taken into account (Figure 4). Three diagnosis categories—hepatitis, cancer, and metabolic diseases—affected 65% of the total cohort, and larger percentages of women than men were in these categories. Women in these categories were less likely to be listed for transplants than were men in the total cohort. As a result, women experienced slightly longer waiting times in the early period than men overall, as shown in Figure 1(a). 

Later waiting times (from listing to transplant) were longer for women, and women were less likely to ever receive a transplant (beta = −0.2165, *P* = .0021; Table 3). Among patients listed for transplantation, the likelihood of death was similar for men and women. The disease-specific interactions did not significantly change the overall effect of gender in the later period.

#### 3.2.2. Race/ethnicity

In the early period (Figures 2(a) and 2(b)), race/ethnicity continued to be important even after adjustment for covariates in the multivariable models. Compared with white patients (the referent group), black patients waited longer and were less likely to be listed for transplantation (beta = −0.7324, *P* < .0001; Table 3), and patients in the Hispanic/Asian/other group had substantially better survival times without listing (beta = –0.29, *P* < .0001). The interaction terms indicate that only black patients with metabolic disorders showed a different pattern from the overall trend in that they tended to be listed sooner and to die sooner (Table 3).

In the later period, the time from listing to transplantation was similar among the racial/ethnic groups and unaffected by the disease-specific interactions (Figure 2(c)). In terms of the competing risk (Figure 2(d)), black patients were more likely than other patients to die on the waiting list without receiving a transplant. The risks of death were affected by disease-specific interactions in that they were lower for Hispanic/Asian/other patients with cancer (beta = −8.938, *P* < .0001; Table 3), higher for Hispanic/Asian/other patients with metabolic disease (beta = 1.197, *P* = .028), lower for patients of unknown race with cancer (beta = −8.964, *P* < .0001), and higher for patients of unknown race with acute liver failure (beta = 1.048, *P* = .01).

#### 3.2.3. Insurance Status

Time to listing was similar for most insurance status groups (Figures 3(a) and 3(b)). The exceptions were patients with combined Medicare/Medicaid coverage, who had longer waiting times (beta = −0.411, *P* = .0043; Table 3), and patients with commercial insurance, who had shorter waiting times (beta = 0.6716, *P* < .0001). Among individuals who were not listed for transplants, the highest risks of dying were in those with combined Medicare/Medicaid (beta = 0.1222, *P* = .0011) and those with Medicaid alone (beta = 0.0809, *P* = .023), whereas the lowest risks of dying were in commercially insured patients (beta = −0.267, *P* < .0001) and uninsured patients (beta = −0.1487, *P* = .018). These trends were also apparent in the disease-specific interactions, where the lowest risks of dying were again in commercially insured patients and uninsured patients (Table 3). 

After listing, there was no variation related to insurance status: both time to transplant and time to death without transplant were similar for all payer groups (Figures 3(a) and 3(b)).

## 4. Discussion

Our analyses of a statewide population-based data set for adults who had liver-related hospitalizations showed that sociodemographics were associated with variation in early waiting times (before being listed for transplant) as well as risk of death. Although the overall experiences were similar for men and women before listing, there was substantial variation related to both race and insurance status. Black patients were less likely to be listed for transplant upon diagnosis. Insurance status also mattered in the early period, in terms of both the likelihood of being listed for transplant and the likelihood of death without ever being listed. Whereas commercially insured patients tended to do better, those covered by Medicare/Medicaid combined were disadvantaged. These patterns may be indicative of disease progression when patients present with symptoms (in this case, when patients are hospitalized), but our analyses did adjust for disease severity at the time of diagnosis.

Once patients are placed on the transplant waiting list, gender appeared more significant as women waited longer to receive a transplant; black patients were more likely to die on the waiting list without a transplant, but insurance status played no role in later waiting time differences. All in all, the timing differences were most pronounced before listing, but were not completely eliminated after listing.

Our study had several limitations that deserve mentioning. First, the study depended on hospitalization data from only one state (Pennsylvania) to identify patients with transplant potential. Second, although the study linked information from 5 participating transplant centers, this information varied across centers in terms of format, detail, and completeness. As a result, we could only explore disparities for 2 periods (diagnosis to listing and then listing to transplant). A more comprehensive analysis of early disparities requires standardizing the data that are collected at these earlier transitions in the transplantation process prior to listing (diagnosis to referral, referral to evaluation, and evaluation to listing). Third, insurance status was based on the index hospitalization only; any potential changes in payer information were not observed. Fourth, the study period predates the MELD scoring system, though it is worth noting that our main finding (i.e., that race/ethnicity and insurance status are associated with variability in early waiting times) refers to stages of the organ allocation process that are unaffected by MELD. Fifth, given the study's retrospective nature and the lack of information about patient preferences for transplantation, we cannot infer causality. 

To our knowledge, our study is the first population-based study of the timing of being listed for transplant services. Previously, we reported differences in the overall likelihood of moving through the allocation and transplant process [15]. The results of the study reported here confirm those earlier findings and provide strong evidence that socioeconomic factors play a role in access to the stages of transplant services in which there is no formal oversight.

With the persistent gap between demand for transplant services and supply of available donor organs, much effort by policymakers and the transplant community is devoted to ensuring the fairness of the transplant system. Where this system is visible and the process is accountable—namely, after individuals are listed by a transplant center—researchers have demonstrated marked improvements in recent years, attributed in part to UNOS oversight and reforms such as the MELD scoring system. Still lacking, however, are centralized data sources to accurately measure the denominator population—that is, the population of all individuals who have end-stage liver disease and are potentially eligible for a transplant. Only with these data can researchers and policymakers measure the true demand for liver-transplant services, assess the fairness of the process, and optimize the allocation of available donor organs.

##  Support and Disclaimers 

This work was supported in part by Grant No. K25 DK002903 from the National Institute for Diabetes and Digestive and Kidney Disorders (NIDDK), which supported C. L. Bryce's Career Development Award during the study period, and Grant No. UL1 RR024153 from the National Center for Research Resources (NCRR) and the National Institutes of Health (NIH) Roadmap for Medical Research. The contents of the work are solely the responsibility of the authors and do not necessarily represent the official view of the NIDDK, NCRR, or NIH. Data used in this paper were supplied by the Pennsylvania Health Care Cost Containment Council (PHC4) and the United Network for Organ Sharing (UNOS). The PHC4 is an independent state agency that has provided data in an effort to further its missions of educating the public and containing health care costs in Pennsylvania. The PHC4, its agents, and staff have made no representation, guarantee, or warranty (express or implied) that the data provided are error-free or that the use of the data will avoid difference of opinion or interpretation, and they bear no responsibility or liability for the results of the analysis, which are solely the opinion of the authors. UNOS is the contractor for the Organ Procurement and Transplantation Network (OPTN). The interpretation and reporting of UNOS data are the responsibility of the authors and in no way should be seen as an official policy of or interpretation by the OPTN or the US government. The authors of the paper have no conflict of interests, including financial interests and relationships and affiliations relevant to the subject of the paper.

## Figures and Tables

**Table 1:** Characteristics of patients with liver-transplant potential*.

	Full cohort of patients	Patients who were listed for a transplant	Patients who received a transplant
Characteristic, no. (%)	*N* = 144,507	*n* = 3,071	*n* = 1,537
Age			
18–39 years	25,779 (17.8)	493 (16.1)	225 (14.6)
40–64 years	60,856 (42.1)	2,365 (77.0)	1,219 (79.3)
≥65 years	57,872 (40.1)	213 (6.9)	93 (6.1)

Gender			
Male	77,885 (53.9)	1,880 (61.2)	987 (64.2)
Female	66,622 (46.1)	1,191 (38.8)	550 (35.8)

Race/ethnicity			
White	103,969 (72.0)	2,267 (73.8)	1,165 (75.8)
Black	19,791 (13.7)	260 (8.5)	114 (7.4)
Other race/ethnicity	6,363 (4.4)	185 (6.0)	87 (5.7)
Unknown	14,384 (10.0)	359 (11.7)	171 (11.1)

Insurance status			
Medicare only	14,315 (9.9)	123 (4.0)	64 (4.2)
Medicare plus Medicaid	7,721 (5.3)	61 (2.0)	24 (1.6)
Medicare plus commercial	40,137 (27.8)	229 (7.5)	107 (7.0)
Medicaid only	24,214 (16.8)	475 (15.5)	219 (14.2)
Commercial only	51,711 (35.8)	2,043 (66.5)	1,049 (68.2)
Uninsured (including self-pay)	4,915 (3.4)	109 (3.5)	59 (3.8)
Unknown	1,494 (1.0)	31 (1.0)	15 (1.0)

Liver disease categories			
Viral hepatitis	28,392 (19.6)	448 (14.6)	211 (13.7)
Alcoholic liver disease	16,301 (11.3)	763 (24.9)	391 (25.4)
Autoimmune disorder	15,652 (10.8)	966 (31.5)	518 (33.7)
Metabolic disease	14,344 (9.9)	15 (0.5)	9 (0.6)
Primary sclerosing cholangitis	6,635 (4.6)	118 (3.8)	56 (3.6)
Cancer	4,754 (3.3)	54 (1.8)	29 (1.9)
Primary biliary cirrhosis	864 (0.6)	80 (2.6)	53 (3.5)
Other chronic disease	46,096 (31.9)	349 (11.4)	156 (10.2)
Acute liver failure	11,469 (7.9)	278 (9.1)	114 (7.4)

Severity of illness at diagnosis			
None	11,985 (8.3)	179 (5.8)	85 (5.5)
Minimal	33,284 (23.0)	509 (16.6)	246 (16.0)
Moderate	39,719 (27.5)	1,142 (37.2)	564 (36.7)
Severe	27,508 (19.0)	746 (24.3)	367 (23.9)
Maximal	3,615 (2.5)	45 (1.5)	16 (1.0)
Unknown	28,396 (19.7)	450 (14.7)	259 (16.9)
Pennsylvania transplant center	N/A	2,758 (89.8)	1,416 (92.1)
Died during study period	57,020 (39.5)	1,027 (33.4)	374 (24.3)

N/A: Not applicable for patients at the diagnosis stage.

*For the Pennsylvania transplant center, *P* = .01. For all other variables, *P* < .001 because of the large sample size.

**Table 2:** Unadjusted time to listing and time to transplantation, controlling for competing risk of death.

BEFORE LISTING									

	Total	Cumulative percent of				Cumulative percent of			
		diagnosed patients listed within:				diagnosed patients who died within:			
		1 year	3 years	5 years	8 years	1 year	3 years	5 years	8 years
All diagnosed patients	144,507	1.30	1.82	2.03	2.12	21.91	30.98	35.71	38.56
Gender									
Male	77,885	1.43	2.05	2.30	2.40	23.52	32.97	37.82	40.66
Female	66,622	1.15	1.55	1.71	1.79	20.01	28.66	33.26	36.11

Race/ethnicity									
White	103,969	1.30	1.85	2.07	2.17	22.87	32.15	37.17	40.20
Black	19,791	0.79	1.11	1.26	1.31	19.17	28.33	32.89	35.60
Hispanic/Asian/other	6,363	1.70	2.52	2.80	2.91	14.27	20.67	23.92	25.65
Unknown	14,384	1.81	2.27	2.44	2.49	22.09	30.76	34.28	36.52

Insurance status									
Medicare only	14,315	0.60	0.78	0.81	0.85	32.90	45.57	51.19	53.83
Medicare + Medicaid	7,721	0.52	0.67	0.76	0.79	29.09	41.73	48.26	51.83
Medicare + commercial	40,137	0.42	0.52	0.57	0.57	33.57	46.69	53.82	58.21
Medicaid only	24,214	1.01	1.66	1.86	1.95	13.13	20.56	24.23	26.74
Commercial only	51,711	2.45	3.36	3.7	3.94	14.14	19.78	22.80	24.65
Uninsured (incl. self-pay)	4,915	1.16	1.87	1.99	2.18	11.23	16.38	19.57	21.02
Unknown	1,494	0.94	1.41	1.81	2.01	12.85	18.41	22.09	26.91

AFTER LISTING									

	Total	Cumulative percent of				Cumulative percent of			
		listed patients transplanted within:				listed patients who died within:			
		1 year	3 years	5 years	8 years	1 year	3 years	5 years	8 years

All listed patients	3,071	36.80	47.80	49.63	50.05	14.23	19.47	20.91	21.26
Gender									
Male	1,880	38.88	50.32	52.18	52.50	13.35	18.40	19.79	20.00
Female	1,191	33.50	43.83	45.59	46.18	15.62	21.16	22.67	23.26

Race/ethnicity									
White	2,267	37.72	48.83	50.90	51.39	13.94	19.19	20.38	20.87
Black	260	33.08	43.08	43.46	43.85	20.39	26.15	28.85	28.85
Hispanic/Asian/other	185	35.68	45.41	47.03	47.03	11.89	17.84	21.08	21.08
Unknown	359	34.26	45.96	47.35	47.63	12.81	17.27	18.38	18.38

Insurance status									
Medicare only	123	40.65	51.22	51.22	52.03	17.07	22.76	24.39	24.39
Medicare + Medicaid	61	34.43	39.34	39.34	39.34	11.48	19.67	21.31	21.31
Medicare + commercial	229	37.99	45.42	46.73	46.73	17.03	24.89	26.64	28.38
Medicaid only	475	33.26	44.00	45.90	46.11	17.26	22.11	24.42	24.84
Commercial only	2,043	37.20	48.80	50.86	51.35	13.22	18.16	19.33	19.53
Uninsured (incl. self-pay)	109	42.20	52.29	53.21	54.13	12.84	17.43	19.27	19.27
Unknown	31	25.81	45.16	48.39	48.39	12.90	19.36	19.36	22.58

**Table 3:** Regressions in the multivariable competing risks model with interaction terms.

	Diagnosis to listing		Diagnosis to death		Listing to transplant		Listing to death	
Variable	Beta	*P*-value	Beta	*P*-value	Beta	*P*-value	Beta	*P*-value
*Age*								
18–39	<ref>				<ref>			
40–64	0.1018	8.70*E* − 02	0.5747	0.00*E* + 00	0.1388	0.11	0.01719	0.89
≥65	−2.181	0.00*E* + 00	0.981	0.00*E* + 00	−0.0751	0.66	0.3803	0.076

*Gender*								
Male	<ref>				<ref>			
Female	0.05282	3.10*E* − 01	−0.2175	0.00*E* + 00	−0.2165	0.0021	0.04415	0.69

*Race/ethnicity*								
White	<ref>				<ref>			
Black	−0.7324	5.90*E* − 12	4.14*E* − 02	1.70*E* − 01	−0.115	0.44	0.5058	7.10*E* − 03
Hispanic/Asian/other	0.1405	2.10*E* − 01	−2.90*E* − 01	1.40*E* − 08	−0.2502	0.11	−0.01805	9.40*E* − 01
Unknown	0.1394	7.30*E* − 02	1.35*E* − 02	6.40*E* − 01	−0.2215	0.045	−0.07929	6.50*E* − 01

*Insurance status*								
Medicare only	<ref>				<ref>			
Medicare plus Medicaid	−0.411	4.30*E* − 02	0.1222	1.10*E* − 03	−0.2997	0.25	−0.2164	0.54
Medicare plus commercial	−0.05088	7.30*E* − 01	−0.004856	8.40*E* − 01	−0.1507	0.41	0.05829	0.8
Medicaid only	−0.1287	3.40*E* − 01	0.08097	2.30*E* − 02	−0.1433	0.37	0.03886	0.87
Commercial only	0.6716	2.60*E* − 08	−0.267	0.00*E* + 00	−0.0757	0.6	−0.1517	0.49
Uninsured (including self-pay)	−0.06924	7.00*E* − 01	−0.1487	1.80*E* − 02	−0.1072	0.61	0.06899	0.82

*Severity of illness at diagnosis*								
None	<ref>				<ref>			
Minimal	0.206	2.00*E* − 02	0.7547	0.00*E* + 00	0.02335	0.85	−0.07078	0.72
Moderate	0.9264	0.00*E* + 00	1.334	0.00*E* + 00	0.02553	0.82	0.002209	0.99
Severe	0.9892	0.00*E* + 00	2.022	0.00*E* + 00	0.01008	0.93	0.1598	0.41
Maximal	0.2154	2.10*E* − 01	2.724	0.00*E* + 00	−0.2741	0.36	1.031	0.0012

*Liver diagnoses (five groups)*								
(1) (PBC, PSC, ALD, and autoimmune)	<ref>				<ref>			
(2) (Viral hepatitis)	−0.7455	1.10*E* − 02	−0.2798	5.60*E* − 07	0.06082	0.59	0.1554	0.38
(3) (Acute liver failure)	−1.111	6.30*E* − 03	0.3353	4.50*E* − 07	−0.3713	0.04	−0.06777	0.79
(4) (Cancer)	−1.959	6.00*E* − 02	0.6301	0.00*E* + 00	−0.2334	0.42	0.1335	0.77
(5) (Metabolic disease, other)	−1.859	1.30*E* − 08	−0.04497	1.40*E* − 01	−0.2278	0.094	0.1449	0.49

*Pennsylvania transplant center*								
No	N/A				<ref>			
Yes					0.3121	0.0021	0.5502	0.0011

*INTERACTION TERMS*								
*Diagnosis group * × * gender*								
2 × Female	−0.1599	1.80*E* − 01	0.006272	8.60*E* − 01	0.05301	0.77	0.2293	0.38
3 × Female	0.459	1.80*E* − 03	0.05483	2.40*E* − 01	0.3382	0.19	0.3079	0.32
4 × Female	−0.9512	8.50*E* − 03	−0.1406	5.60*E* − 03	1.218	0.035	−0.2633	0.77
5 × Female	−0.2384	6.20*E* − 02	0.004479	8.40*E* − 01	0.07122	0.73	−0.03655	0.9

*Diagnosis group * × * race/ethnicity*								
2 × Black	−0.03861	8.30*E* − 01	0.1662	2.80*E* − 04	−0.2849	0.31	−0.2854	0.41
3 × Black	0.243	3.30*E* − 01	−0.06367	3.20*E* − 01	−0.2003	0.64	−0.4171	0.41
4 × Black	0.5954	3.00*E* − 01	0.05434	5.60*E* − 01	0.361	0.73	−0.2571	0.8
5 × Black	0.5146	2.80*E* − 02	0.08273	4.30*E* − 02	−0.3152	0.45	−0.2944	0.53
2 × Hispanic/Asian/other	−0.06936	7.40*E* − 01	0.1438	8.20*E* − 02	0.2924	0.34	−0.6491	0.23
3 × Hispanic/Asian/other	0.3302	3.00*E* − 01	0.121	3.50*E* − 01	0.301	0.6	0.9397	0.071
4 × Hispanic/Asian/other	0.5255	2.50*E* − 01	−0.2266	9.30*E* − 02	0.7187	0.12	−8.938	0
5 × Hispanic/Asian/other	−0.009698	9.80*E* − 01	0.0801	2.80*E* − 01	−0.9032	0.26	1.197	0.028
2 × Unknown	−0.3849	5.30*E* − 02	−0.01653	8.00*E* − 01	0.3819	0.11	−0.8792	0.16
3 × Unknown	0.2203	3.10*E* − 01	0.0593	4.30*E* − 01	−0.8466	0.092	1.048	0.01
4 × Unknown	−0.01568	9.70*E* − 01	−0.08379	3.30*E* − 01	−0.1711	0.8	−8.964	0
5 × Unknown	−0.07788	7.10*E* − 01	0.01727	6.50*E* − 01	0.5868	0.069	−0.2218	0.66

*Diagnosis group * × * insurance status*								
2 × Medicare plus Medicaid	0.01518	9.70*E* − 01	0.0759	3.30*E* − 01
3 × Medicare plus Medicaid	−0.2036	7.80*E* − 01	−0.08408	4.70*E* − 01
4 × Medicare plus Medicaid	−3.869	1.70*E* − 04	−0.1627	2.40*E* − 01
5 × Medicare plus Medicaid	−0.004049	9.90*E* − 01	0.005655	9.10*E* − 01
2 × Medicare plus commercial	0.1845	6.20*E* − 01	0.004062	9.50*E* − 01
3 × Medicare plus commercial	0.4838	3.20*E* − 01	−0.1051	1.70*E* − 01	N/A			
4 × Medicare plus commercial	0.6797	5.50*E* − 01	−0.2542	7.90*E* − 04
5 × Medicare plus commercial	−0.1228	7.50*E* − 01	−0.04907	1.30*E* − 01
2 × Medicaid only	−0.3384	2.90*E* − 01	−0.1179	6.50*E* − 02
3 × Medicaid only	0.1897	6.60*E* − 01	−0.6395	6.20*E* − 15
4 × Medicaid only	0.5077	6.70*E* − 01	0.001579	9.90*E* − 01
5 × Medicaid only	0.2893	4.30*E* − 01	−0.1224	1.20*E* − 02
2 × Commercial only	−0.2162	4.70*E* − 01	−0.2046	9.00*E* − 04				
3 × Commercial only	−0.2264	5.80*E* − 01	−0.274	1.90*E* − 04				
4 × Commercial only	0.6286	5.40*E* − 01	−0.1069	2.00*E* − 01				
5 × Commercial only	−0.2812	4.00*E* − 01	−0.09617	9.70*E* − 03				
2 × Uninsured	−0.2524	5.40*E* − 01	−0.5278	9.30*E* − 06				
3 × Uninsured	−0.7369	2.10*E* − 01	−0.5542	1.10*E* − 05				
4 × Uninsured	1.552	2.00*E* − 01	−0.5514	1.50*E* − 02				
5 × Uninsured	0.4768	3.10*E* − 01	−0.2448	1.10*E* − 02				

Abbreviations: PBC-primary biliary cirrhosis; PSC-primary sclerosing cholangitis; ALD-alcoholic liver disease.

## Abbreviations

DRG: Diagnosis-related group
MEDISGRPS: Medical illness severity grouping system
MELD: Model for end-stage liver disease
PHC4: Pennsylvania Health Care Cost Containment Council
UNOS: United Network for Organ Sharing.
